# *Notes from the Field:* Environmental Contamination from E-cigarette, Cigarette, Cigar, and Cannabis Products at 12 High Schools — San Francisco Bay Area, 2018–2019

**DOI:** 10.15585/mmwr.mm6840a4

**Published:** 2019-10-11

**Authors:** Jeremiah Mock, Yogi H. Hendlin

**Affiliations:** ^1^Institute for Health and Aging, Department of Social and Behavioral Sciences, University of California, San Francisco; ^2^Marin Healthy Youth Partnerships, Larkspur, California; ^3^Dynamics of Inclusive Prosperity Initiative, Erasmus School of Philosophy, Erasmus University Rotterdam, Netherlands; ^4^Environmental Health Initiative, University of California, San Francisco.

The United States is experiencing an epidemic of lung injury associated with youth electronic cigarette (e-cigarette) use, or vaping (*1*); in 2018, 20.8% of U.S. high school students reported currently using e-cigarettes (*1*). E-cigarette products such as Juul, a popular device that delivers nicotine and flavors,* are used by students at schools, including in classrooms and bathrooms.^†^ Use of flavored e-cigarettes by youths has become an increasing concern (*2*). A recent analysis of the National Youth Tobacco Survey showed that among high school students who currently used e-cigarettes, the percentage who used flavored e-cigarettes increased from 65.1% in 2014 to 67.8% in 2018 (*3*). In 2018, 8.1% of high school students currently smoked cigarettes, and 45.7% of those students smoked menthol cigarettes. In addition, 7.6% of high school students currently smoked cigarillos, little cigars, or cigars, 43.6% of whom used flavored varieties of these products (*1*,*3*). Many youths also use cigars to make marijuana blunts (i.e., cigarillos with the tobacco removed and replaced with marijuana) (*4*), and some use manufactured disposable cannabis products (e.g., vape pens, vaporizer cartridges, oils, and concentrates) (*5*). Waste from e-cigarette products can contain plastics, nicotine, heavy metals, other chemical toxins, and hazardous lithium-ion batteries (*6*,*7*). The toxicity of combustible tobacco product waste from cigarettes (e.g., plastic cellulose acetate, nicotine, formaldehyde, lead, and cadmium) is well established (*8*). Cannabis product waste can include plastics, metals, electronic components, and batteries.

A garbology^§^ study of environmental contamination from e-cigarette product waste, combustible tobacco product waste, and cannabis product waste was conducted using a purposively selected, nonrandom sample of 12 public high schools with a total enrollment of 18,831 students in Alameda, Contra Costa, Marin, and San Francisco counties in California. Using 2016 data from the National Center for Education Statistics, researchers stratified schools by the percentages of students from low-income families (i.e., those with students eligible for free or reduced-price lunch).^¶^ At each school, researchers systematically scanned the student parking lots and exterior school perimeter areas once during July 2018–April 2019 to collect all e-cigarette product waste, combustible tobacco product waste, and cannabis product waste found on the ground.

Overall, 893 waste items were collected, including 172 (19%) e-cigarette product waste items (nearly all were Juul or Juul-compatible pods and pod caps) (Table). Almost all Juul or Juul-compatible pods and caps were found at schools with predominantly middle- and upper-income student populations. Among 74 (43%) Juul or Juul-compatible color-coded flavor caps, 73 (99%) were from flavored pods other than tobacco flavor. Overall, 47 (64%) pod caps were from mint-flavored (e.g., Cool Mint) and other menthol-flavored (e.g., Cool Cucumber and Classic Menthol) pods. Additional scans were conducted at one upper-income area school beginning 3 months after Juul Laboratories announced it was removing flavors (except Cool Mint and Classic Menthol) from retail distribution. These additional scans yielded 127 mint, 20 mango, four fruit Juul or Juul-compatible pod caps, and three yellow (banana or mango) Juul-compatible caps.

**TABLE:** Electronic cigarette, combustible tobacco product, and cannabis product waste collected at 12 high schools, by percentage of students from low-income families*^,†^ and other demographic characteristics — San Francisco Bay Area, 2018–2019

Characteristic	Low income*					Middle income*					Upper income*					Total
Public high school no.	1	2	3	4	Subtotals and averages for schools 1–4	5	6	7	8	Subtotals and averages for schools 5–8	9	10	11	12	Subtotals and averages for schools 9–12	1–12
Student population*^,†^	**1,528**	**1,583**	**865**	**1,210**	**5,186**	**2,685**	**1,117**	**1,296**	**3,205**	**8,303**	**1,076**	**1,077**	**1,419**	**1,770**	**5,342**	**18,831**
Students from low-income families (%)	92	88	81	56	79.3	40	32	32	28	33.0	20	7	9	4	10.0	**40.8**
Students learning English as a second language (%)	43	22	37	22	31.0	2	11	11	6	7.5	7	1	2	1	2.8	**13.8**
Female students (%)	51	47	42	50	47.5	59	46	53	51	52.3	46	50	49	49	48.5	**49.4**
**Percentage of students, by race/ethnicity^†,§^**																
Latino or Hispanic	86	27	64	64	60.3	10	40	36	21	26.8	26	11	9	11	14.3	**33.8**
African American or black	5	35	25	1	16.5	2	2	3	19	6.5	3	2	5	1	2.8	**8.6**
White	1	2	2	29	8.5	15	45	50	40	37.5	60	77	69	74	70.0	**38.7**
Asian	6	33	7	5	12.8	65	8	5	9	21.8	5	3	9	7	6.0	**13.5**
American Indian or Alaska Native	<1	<1	<1	<1	<1	<1	1	1	<1	<1	1	<1	<1	<1	<1	**<1**
Hawaiian Native or Pacific Islander	<1	1	1	<1	<1	1	<1	<1	<1	<1	<1	<1	<1	<1	<1	**<1**
Multiracial	<1	2	1	<1	1.0	7	2	5	11	6.3	4	6	8	7	6.3	**4.3**
**Total no. of waste items**	**18**	**71**	**232**	**38**	**359**	**67**	**39**	**12**	**30**	**148**	**15**	**33**	**118**	**220**	**386**	**893**
**Total no. of Juul and Juul-compatible items**	**0**	**0**	**2**	**6**	**8**	**0**	**6**	**3**	**3**	**12**	**3**	**6**	**15**	**128**	**152**	**172**
Juul or Juul-compatible pods	0	0	1	3	4	0	1	0	1	2	0	1	7	33	41	**47**
Juul or Juul-compatible pod black end-caps	0	0	1	3	4	0	0	0	0	0	1	1	4	39	45	**49**
Juul or Juul-compatible Classic Tobacco cap	0	0	0	0	0	0	1	0	0	1	0	0	0	0	0	**1**
Juul or Juul-compatible Virginia Tobacco cap	0	0	0	0	0	0	0	0	0	0	0	0	0	1	1	**1**
Juul or Juul-compatible Cool Mint cap	0	0	0	0	0	0	0	0	2	2	2	4	0	31	37	**39**
Juul or Juul-compatible Mango cap	0	0	0	0	0	0	4	1	**0**	5	0	0	4	10	14	**19**
Juul or Juul-compatible Cool Cucumber cap	0	0	0	0	0	0	0	0	0	0	0	0	0	7	7	**7**
Juul or Juul-compatible Classic Menthol cap	0	0	0	0	0	0	0	0	0	0	0	0	0	1	1	**1**
Juul or Juul-compatible Crème Brulee cap	0	0	0	0	0	0	0	0	0	0	0	0	0	3	3	**3**
Juul or Juul-compatible Fruit Medley cap	0	0	0	0	0	0	0	0	0	0	0	0	0	2	2	**2**
Juul-compatible yellow cap	0	0	0	0	0	0	0	0	0	0	0	0	0	1	1	**1**
Juul Cool Mint 5% 4-pack	0	0	0	0	0	0	0	0	0	0	0	0	0	1	1	**1**
Juul Mango 5% 4-pack	0	0	0	0	0	0	0	1	0	1	0	0	0	0	0	**1**
Juul unknown 4-pack	0	0	0	0	0	0	0	1	0	1	0	0	0	0	0	**1**
**Total no. of little cigar or cigarillo items**	**8**	**26**	**37**	**0**	**71**	**4**	**0**	**3**	**9**	**16**	**0**	**0**	**0**	**0**	**0**	**87**
Little cigar or cigarillo wrappers	7	17	26	0	50	3	0	0	7	10	0	0	0	0	0	**60**
Little cigar or cigarillo mouth pieces or butts	1	9	11	0	21	1	0	3	2	6	0	0	0	0	0	**27**
**Total no. of cigarette butts^¶^**	**8**	**42**	**193**	**32**	**275**	**59**	**33**	**6**	**15**	**113**	**12**	**27**	**103**	**90**	**232**	**620**
Identifiable cigarette butts	8	40	65	29	142	51	25	1	15	92	4	23	83	59	169	**403**
Marlboro menthols	1	6	10	1	18	5	2	0	2	9	0	4	2	5	11	**38**
Newport menthols	1	15	22	1	39	4	0	0	6	10	0	0	1	0	1	**50**
Camel menthols	2	4	9	0	15	0	6	1	2	9	0	3	8	12	23	**47**
All other menthols	0	6	7	0	13	2	3	0	2	7	0	0	11	2	13	**33**
% of menthol among all identifiable butts	50	78	74	7	**60**	22	44	100	80	**38**	0	30	27	32	**28**	**45**
**Total no. of cannabis items**	**2**	**3**	**0**	**0**	**5**	**4**	**0**	**0**	**3**	**7**	**0**	**0**	**0**	**2**	**2**	**14**
Butts (roaches)	1	0	0	0	1	1	0	0	0	1	0	0	0	0	0	**2**
Cartridges/Mouthpieces	1	0	0	0	1	1	0	0	1	2	0	0	0	2	2	**5**
High-potency oil concentrate packaging	0	3	0	0	3	2	0	0	2	4	0	0	0	0	0	**7**

* Low-income families are defined as those with students eligible for free or reduced-price lunch. Stratification of schools is as follows: low income = >50% of students from low-income families; middle income = 25%–50% of students from low-income families; upper income = <25% of students from low-income families.

^†^ National Center for Education Statistics, 2016. https://nces.ed.gov/ccd/schoolsearch/.

^§^ National Center for Education Statistics data are reported as mutually exclusive categories of white, African American or black, Asian, American Indian or Alaska Native, Hawaiian Native or other Pacific Islander, Hispanic or Latino, or multiracial.

^¶^ Total cigarette butts do not equal sum of items because categories overlap.

At four high schools with populations composed predominantly of lower-income African-American and Latino students, eight e-cigarette product waste items were collected, in addition to 71 little cigar or cigarillo plastic wrappers and mouthpieces, 94% of which were from flavored products. No little cigar or cigarillo items were found at schools in upper-income communities.

Across all schools, 620 cigarette butts were collected, including 403 (65%) from recently smoked cigarettes that were identifiable. Among these, 168 (42%) were menthol. At low-, middle-, and upper-income schools, identifiable menthol butts accounted for 60%, 38%, and 28%, respectively, of all identifiable cigarette butts. Fourteen cannabis product waste items were found, including vaporizer pens, cartridges, and packaging from high-potency pineapple- and lemon-flavored cannabis oil concentrate vaporizer cartridges.

E-cigarette waste and combustible tobacco product waste contaminate the Bay Area high schools studied and confirm use of these products by high school students. Cannabis product waste represents an emerging issue. The large proportions of flavored products identified in this study are consistent with findings from other studies showing high prevalence rates of flavored e-cigarette and combustible tobacco product use among U.S. youths. Further research and actions at national, state, and community levels are needed to inform policymaking to reduce youth access to and use of tobacco products, including e-cigarettes, and cannabis products. Youth use of flavored tobacco products, including mint and all other mentholated flavors, is of particular concern. Likewise, measures are needed to eliminate environmental contamination from e-cigarette, combustible tobacco product, and cannabis product waste in and around schools. Schools can engage students in garbology projects to identify existing and new use of these products and to raise awareness about their hazardous health and environmental impacts.

## Footnotes

* Menthol is one of the types of tobacco-product flavoring. https://www.tobaccofreekids.org/assets/factsheets/0394.pdf.

† https://truthinitiative.org/research-resources/emerging-tobacco-products/nearly-1-5-youth-say-they-have-seen-juul-used-school.

§ The ethno-archeological study of a community or cultural group by analyzing its waste.

¶ https://nces.ed.gov/ccd/schoolsearch/.
